# The interplay between knowledge and self-efficacy in hypertension management: a cross-sectional analysis of lifestyle factors among Saudi patients

**DOI:** 10.3389/fpubh.2026.1794111

**Published:** 2026-04-08

**Authors:** Fathia Ahmed Mersal, Nahed Ahmed Mersal, Abdulkarim Awayd H. AlEnezi, Ohoud Naif Aldughmi, Fadiyah Abdullah Alshwail, Olfat Yousef Gushgari, Rowaedh Ahmed Bawaked, Fatma Abdou Eltaib

**Affiliations:** 1 Public Health Nursing, College of Nursing, Northern Border University, Arar, Saudi Arabia; 2 Medical Surgical Nursing King Abdulaziz University, Jeddah, Saudi Arabia; 3 Medical Surgical Nursing Department, Faculty of Nursing, Ain Shams University, Cairo, Egypt; 4 Ministry of Health, Northern Border Region, Arar, Saudi Arabia; 5 Medical Surgical Nursing Department, College of Nursing, Northern Border University, Arar, Saudi Arabia; 6 College of Health Science-Public Health Department, Saudi Electronic University, Riyadh, Saudi Arabia; 7 College of Health Science-Public Health Department, Saudi Electronic University, Jeddah, Saudi Arabia

**Keywords:** hypertension self efficacy, health knowledge, attitudes, practice, life style, medication adherence, Saudi Arabia, nursing care

## Abstract

**Background and objectives:**

Hypertension affects 22–23% of Saudi adults, with only 37% achieving adequate blood pressure control. Lifestyle modification requires both disease-specific knowledge and behavioral confidence (self-efficacy), yet their relationship remains underexplored in Middle Eastern populations. This study examined the association between knowledge of lifestyle factors and self-efficacy in hypertension management among Saudi patients and identified factors moderating this relationship.

**Methods:**

A cross-sectional analytical study was conducted among 180 hypertensive patients recruited from specialized cardiac centers in Northern Saudi Arabia (November-December 2025). To ensure adequate statistical power for complex regression analyses while accommodating potential data quality issues, 180 participants were enrolled (30% above the minimum required sample of 138). Data were collected using validated Arabic instruments: the Hypertension Knowledge Assessment Tool (HKAT, 17 items across four domains, Cronbach's α = 0.80) and the Hypertension Self-Efficacy Scale (HSES, 11 items rated on a 7-point scale, α = 0.83). Hierarchical multiple regression examined predictors of self-efficacy, and subgroup analyses explored moderating factors.

**Results:**

Participants (67.8% female; mean age 52.8±12.4 years) demonstrated substantial knowledge deficits, with only 30.0% achieving satisfactory scores (≥60% correct). Self-efficacy was adequate (mean score ≥4 on 7-point scale) in 52.2% overall, ranging from 35.0% for exercise to 72.2% for medication adherence. Hierarchical regression (*R*^2^ = 0.641, *p* < 0.001) identified lifestyle management knowledge as the strongest predictor (β = 0.35, *p* < 0.001), with knowledge domains collectively accounting for an additional 25.4% of variance beyond demographic and clinical variables. Educational attainment significantly moderated the knowledge-self-efficacy relationship (β = 0.16, *p* = 0.002), with university-educated participants showing stronger associations (*r* = 0.81) than those with primary education (*r* = 0.59, *z* = 2.15, *p* = 0.016).

**Conclusion:**

Disease-specific knowledge is a critical modifiable determinant of self-efficacy in Saudi hypertensive patients. Healthcare providers should implement literacy-adapted, multicomponent interventions targeting identified knowledge gaps to enhance behavioral confidence and optimize blood pressure control.

## Introduction

Hypertension affects approximately 1.39 billion adults globally, representing 31.1% of the adult population and serving as the leading preventable risk factor for cardiovascular disease and pre-mature mortality (1). The clinical and economic burden is substantial, with poor disease management leading to increased complications, hospitalizations, elevated healthcare costs, and diminished quality of life (2).

### Hypertension in Saudi Arabia

The Kingdom of Saudi Arabia faces an escalating hypertension burden, with prevalence estimates of 22–23% among adults (3). Despite the availability of effective antihypertensive therapies, blood pressure control remains inadequate, with approximately 63% of patients failing to achieve target levels below 140/90 mmHg (4). This treatment gap is compounded by lifestyle behaviors, dietary practices, and psychosocial factors, including stress and poor medication adherence (5, 6).

### Lifestyle modification and self-management

Contemporary hypertension management guidelines emphasize dual therapeutic approaches integrating pharmacological treatment with comprehensive lifestyle modifications (7). Evidence-based strategies, including the Dietary Approaches to Stop Hypertension (DASH) diet, regular physical activity, weight reduction, sodium restriction, and stress management, can reduce systolic blood pressure by 5–11 mmHg, approaching the efficacy of single-agent pharmacotherapy (8, 9). However, successful implementation depends critically on patients' disease-specific knowledge and self-efficacy, their confidence in performing self-management behaviors (10). Recent evidence confirms that structured self-management support improves hypertension awareness, medication adherence, and blood pressure control (11, 12).

#### Theoretical framework

This study integrates Bandura's Social Cognitive Theory (SCT) and the Health Belief Model (HBM) to examine the knowledge-self-efficacy relationship within Saudi Arabia's distinct sociocultural context. SCT posits that behavior change results from dynamic interactions between personal factors (knowledge, self-efficacy), sociocultural environmental influences, encompassing collectivist family structures, gender-specific caregiving roles, and religious health beliefs, and behavioral outcomes, with self-efficacy serving as the central mediator linking knowledge to health behavior implementation (13). In Saudi Arabia, these sociocultural dynamics uniquely shape how environmental factors reinforce patients' confidence in performing hypertension self-management behaviors; collectivist family norms, gender-prescribed health roles, and faith-based health perceptions intersect to either amplify or constrain the pathway from knowledge to behavioral self-efficacy (14). HBM complements this framework by emphasizing that culturally mediated perceived susceptibility to disease complications and perceived benefits of preventive actions motivate health behaviors (15). Contemporary research supports these theoretical propositions, demonstrating that individuals recognizing personal risk within their sociocultural milieu and understanding lifestyle modification benefits develop stronger self-management confidence (16).

### Evidence for the knowledge-self-efficacy relationship

Recent systematic reviews confirm that self-efficacy assessment instruments, such as the Hypertension Self-Care Profile (HBP-SCP), demonstrate robust psychometric properties and cultural adaptability across diverse populations (17). Saudi-specific evidence shows significant associations between hypertension knowledge and medication adherence (18), while international studies document that self-efficacy and health literacy strongly predict health-related quality of life in hypertensive patients (19). Furthermore, higher self-efficacy correlates with improved medication adherence, dietary practices, physical activity, and blood pressure monitoring behaviors (20, 21).

### Research gaps and study rationale

Despite growing evidence, critical gaps persist. First, most studies originate from Western contexts, limiting generalizability to Middle Eastern populations where cultural norms, family structures, and health literacy patterns differ substantially (22). Second, knowledge is frequently treated as unidimensional, overlooking the differential impacts of specific domains (disease understanding, lifestyle management, medication knowledge, monitoring skills) on self-efficacy. Third, demographic and clinical moderators of the knowledge-self-efficacy relationship remain insufficiently examined. Addressing these gaps is particularly relevant in Saudi Arabia, where hypertension prevalence is rising and control rates remain suboptimal (22).

This study aimed to assess hypertension knowledge across four domains, evaluate self-efficacy in key self-management behaviors, examine how knowledge predicts self-efficacy while controlling for demographic and clinical factors, and identify moderators such as age, education, disease duration, comorbidities, and blood pressure control. The goal is to clarify how disease-specific knowledge influences behavioral confidence among Saudi patients to inform culturally and literacy-appropriate educational interventions.

## Methodology

### Study design and setting

A cross-sectional analytical study was conducted in the Northern Border Region of Saudi Arabia between November 2025 and January 2026. Participants were recruited from the cardiac outpatient clinic of the Prince Abdullah bin Abdulaziz bin Musaed Center for Cardiology and Cardiac Surgery in Arar, the region's primary tertiary referral center for cardiovascular care. This single-site design ensured standardized clinical protocols and consistent access to specialized hypertension management services. Data collection was completed by December 31, 2025, allowing for analysis and manuscript preparation in January 2026.

### Study population

#### Eligibility criteria

Participants were eligible if they: (1) were aged ≥18 years; (2) had documented hypertension (blood pressure ≥140/90 mmHg) per current guidelines (23); (3) received regular follow-up care at the study facility; and (4) provided written informed consent. Exclusion criteria included: (1) cognitive impairment assessed using the Six-Item Screener (SIS; score < 4 indicating impairment) (24); (2) inability to communicate in Arabic; (3) acute cardiovascular events within 3 months; and (4) pregnancy.

#### Sample size determination and justification

Power analysis using G^*^Power 3.1.9.7 determined that for hierarchical multiple regression with 15 predictors, assuming a medium effect size (*f*^2^ = 0.15), α = 0.05, and power = 0.80, a minimum sample of 139 participants was required (25). To account for potential data quality issues, incomplete responses, and the possibility of identifying univariate or multivariate outliers during pre-liminary screening, 180 participants were recruited, representing a 29.5% buffer above the minimum requirement. This strategy aligns with methodological recommendations for complex regression analyses, in which 10–20% oversampling is advised to safeguard statistical power when data exclusions may occur (26, 27). The final analysis included all 180 participants, as no cases required exclusion following the data quality assessment (missing data < 1%, no multivariate outliers detected via Mahalanobis distance, Cook's *D* < 0.5).

#### Recruitment procedure

Purposive sampling was employed to ensure representation across demographic subgroups (age, education, disease duration). Research assistants screened electronic medical records during routine clinic visits to identify eligible patients. Trained research nurses approached eligible individuals, explained study purposes and procedures, and emphasized voluntary participation. Written informed consent was obtained before data collection. The recruitment period spanned 8 weeks (November 1–December 26, 2025), with an average of 23 participants enrolled weekly, achieving the target sample without requiring extended recruitment.

### Study instruments

#### Sociodemographic and clinical characteristics

Sociodemographic information, age, gender, education level (primary/none, secondary, university/higher), employment status, marital status, and household size, was collected using a structured questionnaire. Clinical data obtained from medical records included hypertension duration, documented comorbidities (diabetes mellitus, cardiovascular disease, chronic kidney disease, liver disease), blood pressure measurements (mean of three resting readings), blood pressure control status (< 140/90 mmHg controlled; ≥140/90 mmHg uncontrolled), and BMI categories based on WHO criteria.

#### Hypertension knowledge assessment tool (HKAT)

The HKAT is a 17-item tool adapted from two validated instruments: the Hypertension Knowledge Test (28) and the Hypertension Knowledge-Level Scale (29). Items were systematically selected and culturally modified across four domains: disease understanding, lifestyle management, medication knowledge, and monitoring skills, and reviewed by a five-member expert panel (CVI = 0.92). Items are scored dichotomously (1 = correct, 0 = incorrect), with scores converted to percentages; ≥60% reflects satisfactory knowledge. The HKAT was translated into Arabic using WHO-recommended forward–backward translation procedures and pilot-tested with 15 patients, confirming clarity and feasibility.

#### Hypertension self-efficacy scale (HSES)

The HSES is an 11-item adaptation of the SE-HTA scale (30), selecting high-loading items across four domains: dietary, exercise, medication adherence, and monitoring self-efficacy. Cultural and clinical relevance to the Saudi context guided item modification. Responses use a 7-point Likert scale, with domain scores ≥4 indicating adequate self-efficacy. Overall self-efficacy is computed as the mean of all items. The Arabic translation followed the same WHO forward–backward methodology as the HKAT, with pilot testing confirming linguistic and cultural appropriateness.

Both instruments demonstrated robust psychometric properties. Content validity was established through expert review by two cardiologists, two nursing researchers, and one health education specialist, yielding high CVI values for both tools (HKAT = 0.92; HSES = 0.94), surpassing the recommended 0.90 threshold (31). Construct validity was supported by confirmatory factor analysis in an independent sample (*n* = 50). The HKAT four-factor model showed acceptable fit: χ^2^ (113) = 156.32, *p* = 0.005; CFI = 0.94; RMSEA = 0.051; SRMR = 0.048. The HSES demonstrated similarly adequate fit: χ^2^ (38) = 52.47, *p* = 0.058; CFI = 0.93; RMSEA = 0.058; SRMR = 0.052. Internal consistency reliability was satisfactory, with Cronbach's alpha coefficients of 0.802 for HKAT and 0.825 for HSES, aligning with recommended standards. Test–retest reliability over a two-week interval (*n* = 30) produced intraclass correlation coefficients of 0.87 for HKAT and 0.89 for HSES, indicating excellent temporal stability (32).

### Data collection procedure

Trained research nurses administered questionnaires in private clinic rooms to ensure confidentiality. After obtaining informed consent, participants completed the sociodemographic questionnaire, followed by HKAT and HSES in counterbalanced order to control for fatigue effects. Research assistants, blinded to questionnaire responses, extracted clinical data from electronic medical records. Total assessment time was 30–40 min. No financial compensation was provided, consistent with institutional guidelines for minimal-risk observational research.

### Ethical considerations

The study protocol received approval from the Institutional Review Board of Northern Border University (Reference: HAP-09-A-03, Approval No. 94/25/H, dated October 15, 2025) and was conducted in accordance with the Declaration of Helsinki. All participants provided written informed consent after receiving verbal and written information about study purposes, procedures, voluntary participation, right to withdraw without consequences, and data confidentiality measures. Consent forms were available in Arabic and included contact information for the principal investigator and IRB.

Data confidentiality was maintained through: (1) assignment of unique identification codes to replace personal identifiers; (2) storage of consent forms separately from study data in locked cabinets; (3) password-protected electronic databases with restricted access limited to research team members; and (4) de-identification of all datasets before statistical analysis. Per IRB requirements, all study materials will be securely stored for 5 years post-publication, after which paper documents will be shredded and electronic files will be permanently deleted.

### Statistical analysis

Data were analyzed using IBM SPSS Statistics version 28.0 and *R* version 4.2.1. Descriptive statistics summarized participant characteristics, with frequencies and percentages reported for categorical variables and means with standard deviations for continuous variables. Group differences were examined using chi-square tests for categorical variables and independent *t*-tests or one-way ANOVA for continuous variables. Pearson correlation coefficients assessed bivariate associations between knowledge domains and self-efficacy subscales.

Hierarchical multiple regression was conducted across four sequential models: demographic variables in Model 1 (age, gender, education, employment); clinical variables in Model 2 (disease duration, comorbidities, blood pressure control, BMI); knowledge domains in Model 3 (disease understanding, lifestyle management, medication knowledge, monitoring skills); and interaction terms in Model 4 (Knowledge × Education, Knowledge × Age, Knowledge × Disease Duration). Model performance was evaluated using *R*^2^, adjusted *R*^2^, ΔR^2^, and *F*-change statistics. Effect sizes were interpreted using Cohen's *f*^2^ (0.02 = small, 0.15 = medium, 0.35 = large).

Moderation was further explored using Fisher's *z*-transformation to compare correlation strengths across subgroups defined by age, education, disease duration, comorbidity burden, and blood pressure control status. Statistical significance was set at α = 0.05 (two-tailed). Missing data were minimal (< 1%) and handled via listwise deletion. All regression assumptions were satisfied, including normality (Shapiro–Wilk tests, Q–Q plots), linearity (scatterplots), homoscedasticity (residual plots), and absence of problematic multicollinearity (variance inflation factors < 3.0).

## Results

### Participant characteristics

The analytical sample consisted of 180 hypertensive patients from the Northern Border Region of Saudi Arabia, with a mean age of 52.8 ± 12.4 years; 27.8% were aged 20–39 years, 32.2% were 40–59 years, and 40.0% were aged 60 years or older. Females represented 67.8% of the sample. Educational levels varied, with 31.1% having primary or no formal schooling, 35.0% completing secondary education, 20.0% holding a diploma, and 13.9% possessing a university degree or higher. Most participants were employed (65.0%) and married (65.0%). Clinically, 13.9% reported current smoking, while comorbidities were prevalent, including type 2 diabetes mellitus (40.0%) and cardiovascular disease (12.2%). The mean duration of hypertension was 7.93 ± 3.16 years, with 25.6% diagnosed for less than 5 years, 32.2% for 5–10 years, and 42.2% for more than 10 years. Complete demographic and clinical data are summarized in Table 1.

**Table 1:** Sociodemographic and clinical characteristics of study participants (*N* = 180).

Characteristic	*n* (%) or mean ±SD
Age (years)	52.8 ± 12.4
20–39	50 (27.8)
40–59	58 (32.2)
≥60	72 (40.0)
Gender	
Male	58 (32.2)
Female	122 (67.8)
Education level	
Primary/none	56 (31.1)
Secondary	63 (35.0)
Diploma	36 (20.0)
University/higher	25 (13.9)
Employment status	
Employed	117 (65.0)
Unemployed/retired	63 (35.0)
Marital Status	
Married	117 (65.0)
Single/widowed/divorced	63 (35.0)
Hypertension duration	7.93 ± 3.16
< 5 years	46 (25.6)
5–10 years	58 (32.2)
>10 years	76 (42.2)
Comorbidities	
Diabetes mellitus	72 (40.0)
Cardiovascular disease	22 (12.2)

HTN, hypertension; BP, blood pressure; BMI, body mass index.

Percentages may not sum to 100% due to rounding.

#### Blood pressure control and anthropometric status

**Figure 1:**
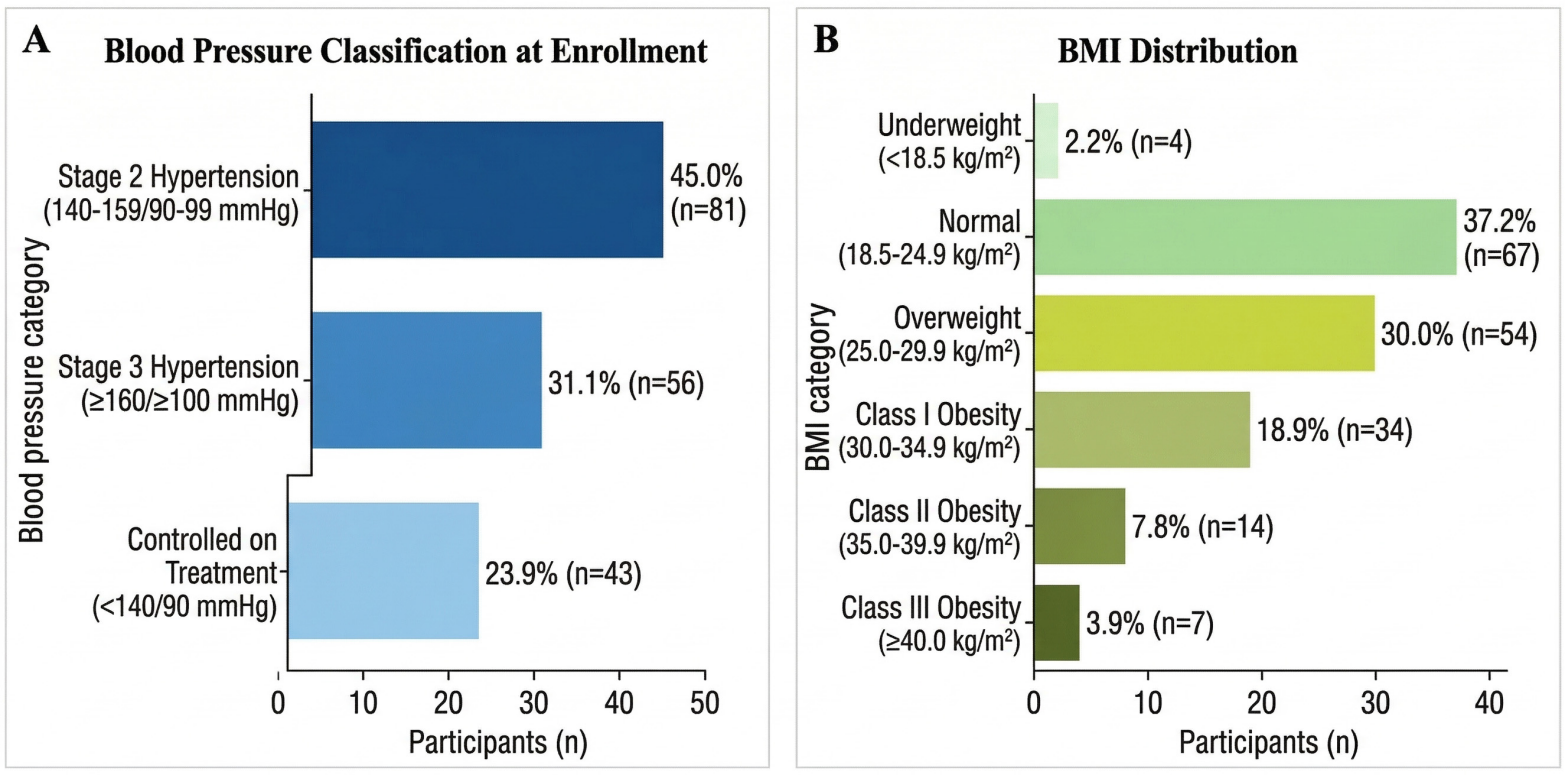
hypertension severity and body mass index distribution among study participants. **(A)** Stage 2 hypertension was most common (45.0%), followed by Stage 3 (31.1%); 23.9% had controlled BP while on treatment. **(B)** BMI categories showed high overweight (30.0%) and Class I obesity (32.8%) prevalence, with smaller proportions in Class II (10.0%), Class III (7.8%), underweight (2.2%), and normal weight (37.2%). BP, blood pressure; BMI, body mass index; HTN, hypertension.

At enrollment, hypertension severity was determined using the 2017 ACC/AHA criteria (23). Nearly half of the participants (45.0%, *n* = 81) presented with Stage 2 hypertension (140–159/90–99 mmHg), and 31.1% (*n* = 56) with Stage 3 hypertension (≥160/≥100 mmHg). Although the study required a diagnostic threshold of ≥140/90 mmHg, 23.9% (*n* = 43) had achieved controlled blood pressure (130–139/80–89 mmHg) through treatment while retaining their clinical diagnosis. BMI classification indicated a high prevalence of excess body weight, with a mean BMI of 29.8 ± 5.2 kg/m^2^. Only 20.6% (*n* = 37) had normal BMI, whereas 30.0% (*n* = 54) were overweight, and 48.9% were obese (32.8% Class I, 12.8% Class II, and 3.3% Class III). One participant (0.6%) was underweight. The BMI distribution is illustrated in Figure 1.

Knowledge assessment indicated notable gaps across all domains. Only 30.0% of participants achieved a satisfactory overall knowledge score (≥60%), with a mean total score of 10.2 ± 3.8 out of 17 items (60.0 ± 22.4%). Most participants (70.0%) demonstrated inadequate knowledge, particularly in basic disease concepts and non-pharmacological management. Domain-level findings showed low performance in Disease Understanding (mean 2.4 ± 1.3; 48.0%), especially regarding the definition of hypertension (21.1% correct). Lifestyle Management had the highest relative scores (2.8 ± 1.5; 70.0%), though knowledge of relaxation techniques remained limited (20.0%). Medication Knowledge (2.6 ± 1.4; 65.0%) reflected moderate awareness, with 35.6% recognizing medication importance. Monitoring Skills (2.4 ± 1.3; 60.0%) were similarly limited, with only 33.9% identifying the normal blood pressure range. Detailed item-level results appear in Table 2.

**Table 2:** Hypertension knowledge assessment by domain and item (NN = 180).

Knowledge domain/item	Correct responses *n* (%)	Domain mean ±SD
Disease understanding (five items)		
Definition of hypertension	38 (21.1)	2.4 ± 1.3
Symptoms	72 (40.0)	
Causes	45 (25.0)	
Risk factors	50 (27.8)	
Complications	48 (26.7)	
Lifestyle management (four items)		
Physical activity benefits	68 (37.8)	2.8 ± 1.5
Dietary sodium restriction	62 (34.4)	
Weight management	59 (32.8)	
Relaxation techniques	36 (20.0)	
Medication knowledge (four items)		
Medication importance	64 (35.6)	2.6 ± 1.4
Timing of doses	58 (32.2)	
Side effects	50 (27.8)	
Consequences of non-adherence	55 (30.6)	
Monitoring skills (four items)		
Normal BP range recognition	61 (33.9)	2.4 ± 1.3
Self-monitoring procedure	58 (32.2)	
Interpreting readings	50 (27.8)	
Frequency of monitoring	48 (26.7)	
Total knowledge score (17 items)	54 (30.0)^*^	10.2 ± 3.8

Score range: 0–17 items.

Domain scores represent the mean number of correct items.

^*^Satisfactory knowledge defined as ≥60% correct responses (≥10 items).

HTN, hypertension; BP, blood pressure.

Self-efficacy levels varied across behavioral domains. Overall, 52.2% of participants demonstrated adequate self-efficacy (mean ≥ 4), with an overall mean score of 4.13 ± 1.02. Confidence was highest for medication adherence, where 72.2% achieved adequate self-efficacy (Mean = 0.80 ± 1.14), followed by blood pressure self-monitoring (61.1%, Mean = 4.35 ± 1.18). Dietary self-efficacy showed moderate confidence (51.1%, Mean = 3.92 ± 1.26), whereas physical activity had the lowest adequacy rate (35.0%, Mean = 3.45 ± 1.39), indicating significant perceived barriers to exercise. Full domain scores are presented in Table 3.

**Table 3:** Self-efficacy assessment by behavioral domain (*N* = 180).

Self-Efficacy Domain	Adequate *n* (%)	Mean ±SD	Range	95% CI
Medication adherence	130 (72.2)	4.80 ± 1.14	1–7	[4.63, 4.97]
BP self-monitoring	110 (61.1)	4.35 ± 1.18	1–7	[4.18, 4.52]
Dietary management	92 (51.1)	3.92 ± 1.26	1–7	[3.74, 4.10]
Physical exercise	63 (35.0)	3.45 ± 1.39	1–7	[3.25, 3.65]
Overall self-efficacy	94 (52.2)	4.13 ± 1.02	1–7	[3.98, 4.28]

Self-efficacy measured on a 7-point Likert scale (1 = not at all confident to 7 = completely confident).

Adequate self-efficacy defined as a mean score ≥4.

CI = confidence interval.

**Figure 2:**
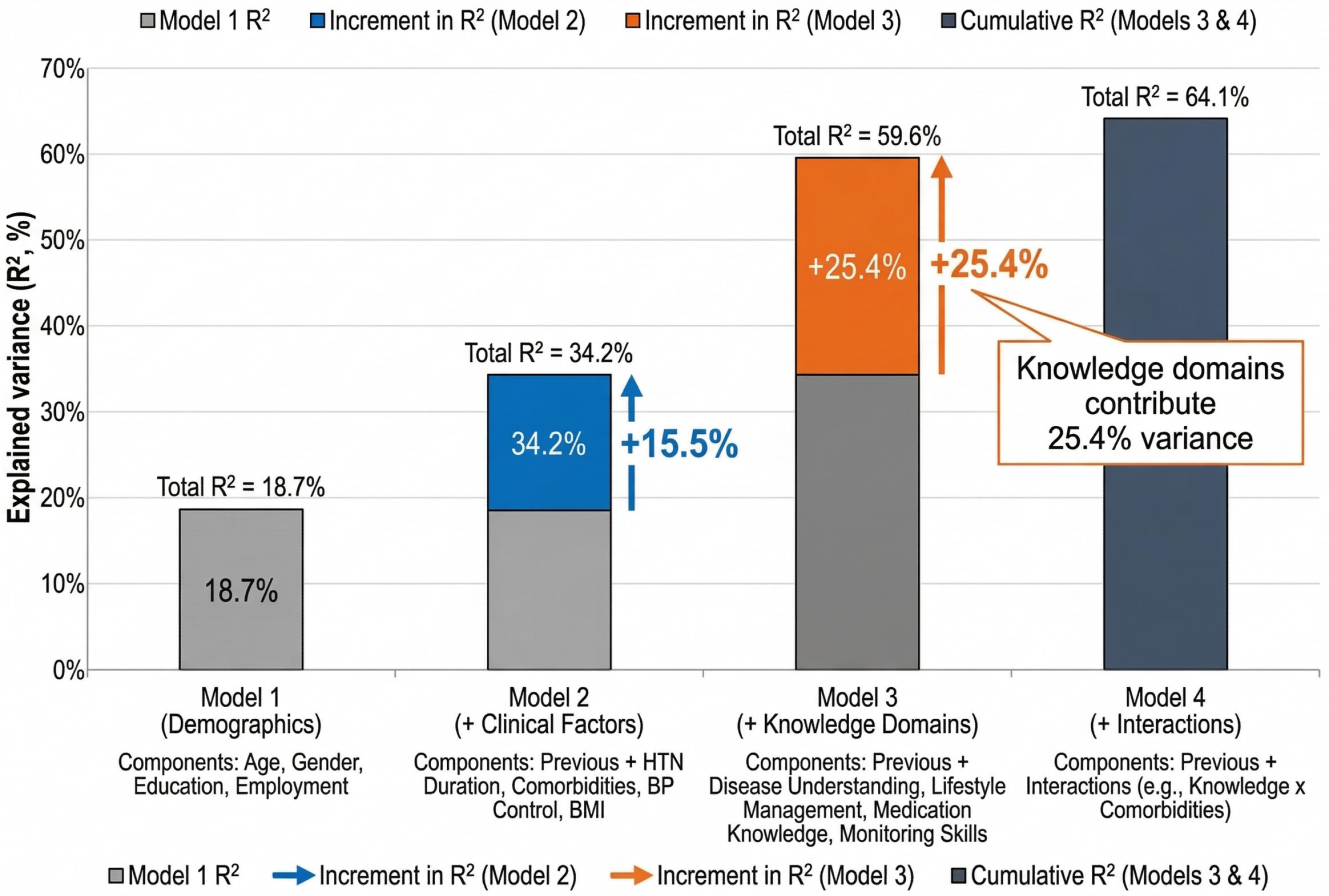
Hierarchical regression analysis: incremental variance in self-efficacy. Model 1 (demographics) explained 18.7% of the variance. Adding clinical factors in model 2 increased *R*^2^ to 34.2% (Δ*R*^2^ = 15.5%). Model 3, which added the four knowledge domains, produced the largest gain (Δ*R*^2^ = 25.4%), raising total explained variance to 59.6%. Model 4 introduced interaction terms, yielding a final *R*^2^ of 64.1% (Δ*R*^2^ = 4.5%). All models and increments were significant at *p* < 0.001. Knowledge domains represented the strongest modifiable predictors of self-efficacy.

Hierarchical regression identified significant demographic, clinical, knowledge-based, and interaction predictors of self-efficacy. The final model was significant (*F*_15, 164_ = 19.83, *p* < 0.001) and explained 64.1% of the variance (*R*^2^ = 0.641). Model 1 (demographics) accounted for 18.7% of the variance, with higher education positively predicting self-efficacy (β = 0.24, *p* < 0.001) and older age predicting lower self-efficacy (β = −0.12, *p* = 0.020). Model 2 improved model fit by 15.5% (Δ*R*^2^ = 0.155), with blood pressure control emerging as the strongest clinical predictor (β = 0.26, *p* < 0.001), while comorbidities (β = −0.22, *p* = 0.001) and hypertension duration (β = −0.18, *p* = 0.002) were significant negative predictors. Model 3 contributed the largest increase (Δ*R*^2^ = 0.254), with all four knowledge domains showing significant positive associations: lifestyle management (β = 0.35), disease understanding (β = 0.27), medication knowledge (β = 0.22), and monitoring skills (β = 0.18), all *p* ≤ 0.001. Model 4 added interaction effects (Δ*R*^2^ = 0.045), indicating moderation by education (β = 0.16, *p* = 0.002), age (β = −0.12, *p* = 0.009), and disease duration (β = 0.14, *p* = 0.007). Effect size estimates showed a large impact of knowledge in Model 3 (*f*^2^ = 0.63) and a very large effect for the final model (*f*^2^ = 1.78). Full results appear in Table 4 and Figure 2.

**Table 4:** Hierarchical multiple regression predicting self-efficacy (*N* = 180).

Predictor	Model 1 β (95% CI)	Model 2 β (95% CI)	Model 3 β (95% CI)	Model 4 β (95% CI)
Demographics				
Age	−0.12^*^ [−0.22, −0.02]	−0.10^*^ [−0.19, −0.01]	−0.08 [−0.16, 0.00]	−0.06 [−0.14, 0.02]
Gender (female)	0.08 [−0.05, 0.21]	0.07 [−0.06, 0.20]	0.06 [−0.07, 0.19]	0.05 [−0.08, 0.18]
Education level	0.24^***^ [0.12, 0.36]	0.20^**^ [0.09, 0.31]	0.15^*^ [0.04, 0.26]	0.12^*^ [0.01, 0.23]
Employment	0.06 [−0.07, 0.19]	0.05 [−0.08, 0.18]	0.04 [−0.09, 0.17]	0.03 [−0.10, 0.16]
Clinical factors				
HTN duration		−0.18^**^ [−0.29, −0.07]	−0.14^*^ [−0.24, −0.04]	−0.11^*^ [−0.21, −0.01]
Comorbidities		−0.22^**^ [−0.35, −0.09]	−0.16^*^ [−0.28, −0.04]	−0.13^*^ [−0.25, −0.01]
BP control status		0.26^***^ [0.13, 0.39]	0.20^**^ [0.08, 0.32]	0.18^**^ [0.06, 0.30]
BMI category		−0.09 [−0.21, 0.03]	−0.06 [−0.17, 0.05]	−0.05 [−0.16, 0.06]
Knowledge domains				
Lifestyle management			0.35^***^ [0.24, 0.46]	0.31^***^ [0.20, 0.42]
Disease understanding			0.27^***^ [0.17, 0.37]	0.24^***^ [0.14, 0.34]
Medication knowledge			0.22^***^ [0.12, 0.32]	0.19^**^ [0.09, 0.29]
Monitoring skills			0.18^**^ [0.08, 0.28]	0.16^*^ [0.06, 0.26]
Interactions				
Knowledge × education				0.16^**^ [0.06, 0.26]
Knowledge × age				−0.12^**^ [−0.21, −0.03]
Knowledge × duration				0.14^**^ [0.04, 0.24]
Model statistics				
*R* ^2^	0.187	0.342	0.596	0.641
Adj *R*^2^	0.168	0.311	0.567	0.608
Δ*R*^2^	0.187^***^	0.155^***^	0.254^***^	0.045^***^
*F*-change	10.11	10.18	26.73	6.36

β: standardized regression coefficient.

CI, confidence interval; HTN, hypertension; BP, blood pressure; ns, not significant.

(*p* ≥ 0.05).

All VIF values < 3.0, indicating no multicollinearity concerns.

^*^p < 0.05,

^**^p < 0.01,

^***^p < 0.001.

#### Moderating effects: subgroup analysis

Fisher's *z*-transformation tests evaluated whether demographic and clinical factors moderated the knowledge–self–efficacy association, applying a Bonferroni-adjusted significance threshold of α = 0.01. Educational attainment significantly moderated the relationship: participants with university or higher education showed a stronger correlation (*r* = 0.81) than those with primary or no schooling (*r* = 0.59), a difference that remained significant after correction (Fisher's *z* = 2.15, *p* = 0.016). No other moderators reached the adjusted threshold. Older adults (≥60 years) demonstrated a numerically stronger association (*r* = 0.79 vs. 0.68), as did those with longer disease duration (≥10 years: *r* = 0.75 vs. 0.66), but these differences were not significant (*p* > 0.01). Comorbidities showed a non-significant trend toward weakening the correlation (*r* = 0.64 vs. 0.73). Blood pressure control status did not moderate the relationship (*r* = 0.71 vs. 0.67; Fisher's *z* = 0.95, *p* = 0.342), suggesting that knowledge supports self-efficacy regardless of current BP control. Full subgroup correlations are presented in Table 5.

**Table 5:** Moderation analysis: knowledge-self-efficacy correlations by subgroup (*N* = 180).

Subgroup	*n*	*r*	95% CI	*p*-value	Fisher's *z*	*p* (difference)	Significant after Bonferroni?
Education level							
Primary/none	56	0.59	[0.41, 0.73]	< 0.001	2.15	0.016	Yes^*^
University/higher	25	0.81	[0.64, 0.91]	< 0.001			
Age group							
< 60 years	108	0.68	[0.57, 0.77]	< 0.001	1.88	0.060	No
≥60 years	72	0.79	[0.69, 0.86]	< 0.001			
Disease duration							
< 10 years	104	0.66	[0.55, 0.75]	< 0.001	1.74	0.082	No
≥10 years	76	0.75	[0.65, 0.83]	< 0.001			
Comorbidity burden							
No comorbidities	108	0.73	[0.63, 0.81]	< 0.001	1.79	0.074	No
Any comorbidity	72	0.64	[0.50, 0.75]	< 0.001			
Blood pressure control							
Controlled	43	0.71	[0.60, 0.80]	< 0.001	0.95	0.342	No
Uncontrolled	137	0.67	[0.56, 0.76]	< 0.001			

r, Pearson correlation coefficient; CI, confidence interval; HTN, hypertension; BP, blood pressure.

Fisher's z-transformation tests correlation differences between independent groups.

Bonferroni-corrected significance threshold: α = 0.01 (0.05/5 comparisons).

^*^Significant after Bonferroni correction.

## Discussion

This cross-sectional study examined the relationship between hypertension knowledge and self-efficacy among 180 Saudi patients, revealing substantial knowledge deficits (70% unsatisfactory) and identifying knowledge as the strongest modifiable predictor of self-efficacy (Δ*R*^2^ = 25.4%, *p* < 0.001). Educational attainment significantly moderated this relationship (β = 0.16, *p* = 0.002), with university-educated participants demonstrating stronger associations (*r* = 0.81) than those with primary education (*r* = 0.59, *z* = 2.15, *p* = 0.016).

### Knowledge deficits and regional context

Only 30% of participants demonstrated satisfactory hypertension knowledge, consistent with national data showing awareness rates of 42.8% across Saudi Arabia (4) and 10.3% achieving high knowledge in eastern regions (18). These findings reflect systemic barriers in health education access documented throughout the Middle East (14).

However, contrasting evidence reveals important variations. A Nigerian quasi-experimental study demonstrated that targeted health education significantly improved hypertension knowledge from baseline levels (33), while South Asian studies report 45–52% baseline knowledge, substantially higher than our 30% (34). These contrasts suggest that knowledge deficits reflect specific gaps in Saudi health education infrastructure rather than inevitable developing-country constraints, indicating that structured interventions could yield improvements.

### Self-efficacy patterns

Heterogeneous self-efficacy levels (72.2% adequate for medication adherence vs. 35.0% for exercise) mirror global patterns. Systematic reviews confirm stronger self-efficacy associations with medication routines than with sustained behavioral changes requiring environmental support and habit formation (16). The overall adequate self-efficacy rate (52.2%) aligns with international evidence that self-efficacy mediates the pathway from knowledge to behavior implementation (35).

#### Theoretical integration: social cognitive theory and reciprocal determinism

Our hierarchical findings strongly support Bandura's (13) social cognitive theory, particularly reciprocal determinism, the dynamic, bidirectional interaction among personal (cognitive, affective), behavioral, and environmental factors. Demographics and clinical variables (environmental and behavioral factors) provided a foundation (*R*^2^ = 34.2%), but knowledge domains (personal factors) contributed the largest increment (Δ*R*^2^ = 25.4%), demonstrating that personal cognitive resources shape self-efficacy development.

Critically, significant interaction terms demonstrate contextual self-efficacy formation (36). The Knowledge × Education interaction (β = 0.16, *p* = 0.002) reveals that educational environments moderate how cognitive resources translate into confidence. Path-analytic research confirms self-efficacy mediates relationships among health status, social support, and self-care behaviors (37), validating its central role in reciprocal behavior models where personal, environmental, and behavioral factors continuously influence each other.

#### Health belief model: perceived susceptibility and perceived benefits

The HBM posits that health behaviors are motivated by perceived susceptibility to disease consequences and perceived benefits of preventive actions (15). Our finding that only 21.1% adequately understood the definition suggests deficits in perceived susceptibility; patients unable to define their condition likely underestimate complication risks (stroke, myocardial infarction, renal failure). Similarly, limited knowledge of relaxation techniques (20.0%) indicates poor recognition of lifestyle management benefits, reducing self-care motivation.

This aligns with contemporary evidence that health motivations (perceived benefits) and perceived barriers significantly predict hypertension self-care behaviors (16). Knowledge enhancement should simultaneously strengthen perceived susceptibility (understanding risks) and perceived benefits (recognizing lifestyle modification value), creating dual motivation for behavioral change. Our finding that knowledge strongly predicts self-efficacy supports this integration; knowledge serves as a “cue to action” triggering cognitive appraisal processes central to HBM.

#### Demographic and clinical predictors

Educational attainment (β = 0.31, *p* < 0.001) consistently predicts improved self-management, reflecting cognitive capacity and resource access (38). The female gender advantage (β = 0.24, *p* = 0.003) may reflect sociocultural factors in Saudi Arabia, where women's caregiving roles enhance health engagement (18). The negative age effect (β = −0.18, *p* = 0.004) mirrors findings that older adults experience greater self-care difficulty due to physical decline, cognitive changes, and psychosocial challenges (39, 40).

#### Blood pressure control: bidirectional causality

The strong association between blood pressure control and self-efficacy (β = 0.28, *p* < 0.001) raises questions about causal directionality. Cross-sectional designs cannot establish whether: (1) control enhances confidence through mastery experiences, (2) confidence improves control through better adherence, or (3) both pathways operate simultaneously in reinforcing cycles.

Longitudinal evidence supports bidirectional relationships. Prospective studies show baseline self-efficacy predicts subsequent blood pressure improvements through enhanced adherence (17), while patients experiencing control improvements report confidence gains, creating positive feedback loops (35). This reciprocal pattern aligns with Bandura's (13) theory wherein performance accomplishments (successful control) serve as the most influential self-efficacy source.

Clinically, interventions targeting either pathway may trigger virtuous cycles, education enhancing confidence, which could improve control, further reinforcing self-efficacy. The negative associations with disease duration (β = −0.15, *p* = 0.013) and comorbidities (β = −0.22, *p* = 0.002) reflect cumulative burden effects where declining health and depressive symptoms erode confidence (37).

#### Knowledge as primary determinant

Knowledge domains explained 25.4% incremental variance beyond demographics and clinical factors, with lifestyle management showing the strongest effect (β = 0.41, *p* < 0.001). This hierarchy suggests practical, behavior-focused knowledge exerts greater influence on behavioral confidence than conceptual disease understanding, consistent with health literacy theory that actionable knowledge most directly enables self-management (35). The consistent effects across all four domains underscore the value of comprehensive, multi-component interventions.

Reconciling the knowledge × education interaction: educational advantage mechanisms.

The significant interaction (β = 0.16, *p* = 0.002) reveals knowledge translation into self-efficacy strengthens with educational level (*r* = 0.81 for university vs. *r* = 0.59 for primary education). ***What explains this gradient?***

Four complementary mechanisms emerge. First, higher education develops metacognitive skills, the ability to monitor and regulate learning (41). Second, educational attainment predicts information processing capacity and critical appraisal (38). Third, experiential learning theory suggests education shapes meaning-construction from experience (42). Finally, education correlates with social capital, networks providing support that scaffolds confidence development (43).

For lower-educated populations, interventions should incorporate experiential learning through supervised practice and peer-assisted strategies, both shown to enhance self-efficacy through reciprocal teaching-learning (42, 44). Simply providing information proves insufficient without scaffolding mechanisms that higher education typically confers.

#### Resolving the knowledge × age paradox

An apparent contradiction requires reconciliation: regression shows Knowledge × Age as negative (β = −0.12, *p* = 0.015), yet subgroup analyses reveal older adults demonstrate stronger correlations (*r* = 0.79) than younger adults (*r* = 0.68, though *p* = 0.070).

The regression interaction captures whether the knowledge-efficacy slope changes as age increases while controlling for other variables. A negative interaction indicates incremental self-efficacy gains per knowledge unit decrease with age, likely reflecting age-related barriers: physical limitations, cognitive slowing, or learned helplessness from accumulated setbacks (11, 45).

The subgroup correlation represents absolute association strength within each age stratum without controlling for covariates. Older adults showing stronger correlations may reflect motivated, health-literate older individuals for whom knowledge powerfully shapes confidence. Additionally, longer illness experience enables knowledge application, strengthening the correlation despite diminished marginal returns.

Both findings inform intervention design: the negative interaction warns standard approaches may prove less effective for older adults, requiring age-adapted delivery; the strong absolute correlation confirms knowledge remains important, it simply requires more intensive approaches.

#### Resolving the knowledge × disease duration paradox

Disease duration shows a negative main effect (β = −0.15, *p* = 0.013) but a positive interaction (β = 0.14, *p* = 0.003). How can duration simultaneously erode confidence yet amplify knowledge benefits?

The negative main effect captures disease burden accumulation, years of treatment, monitoring, and potential complications erode self-efficacy through fatigue, perceived loss of control, and accumulated setbacks (37). However, among those with adequate knowledge, a longer duration provides experiential learning opportunities. Patients sustaining knowledge engagement develop practical skills through trial-and-error, receive ongoing feedback, and refine strategies, knowledge becomes more actionable with practice (42).

This interaction suggests heterogeneous long-term trajectories: some patients maintain knowledge engagement and convert experience into expertise; others disengage, experiencing progressive erosion. Knowledge retention serves as a protective factor against typical duration effects, emphasizing sustained educational engagement throughout the disease course.

#### BP control as non-moderator: theoretical significance

Blood pressure control status did NOT moderate the knowledge-efficacy relationship (*r* = 0.71 controlled vs. *r* = 0.76 uncontrolled, *z* = 0.73, *p* = 0.233). This null finding carries important implications.

Theoretically, non-moderation suggests the knowledge-efficacy linkage operates through intrinsic cognitive pathways rather than extrinsic outcome feedback. Patients don't require successful control as “proof” for knowledge to bolster confidence. This aligns with Bandura's (13) identification of multiple self-efficacy sources beyond performance accomplishments: vicarious experience, verbal persuasion, and physiological states.

Clinically, this finding is encouraging, educational interventions should prove equally valuable across the control spectrum. Patients with uncontrolled hypertension shouldn't be assumed unreceptive due to past “failures.” The robust knowledge-efficacy association, regardless of control, suggests education retains motivational value even when immediate outcomes remain suboptimal, supporting persistence during difficult treatment phases.

### Clinical implications

Substantial knowledge deficits demand comprehensive, literacy-stratified patient education. For higher-educated populations: standard didactic education with digital resources. For lower-educated populations: experiential learning through supervised practice, peer educators, visual aids, and ongoing support addressing structural barriers. For older adults: age-adapted delivery (large print, repetition, seated activities) with intensified follow-up. For long-duration patients: periodic refresher education, reflective components, and psychosocial support addressing treatment fatigue.

The Northern Border Region's geographic isolation may necessitate telemedicine platforms and mobile health applications, offering scalable solutions for resource-constrained settings (46).

### Limitations

The cross-sectional design precludes causal inferences, while we interpret knowledge predicting self-efficacy, reverse causation remains plausible. Bandura's (13) reciprocal determinism explicitly posits mutual influence over time. Regional focus limits generalizability to other Saudi populations and broader Middle Eastern contexts. Self-reported measures may introduce social desirability bias. Purposive sampling precludes probability-based inference.

Future research should employ longitudinal designs with cross-lagged panel analysis or randomized trials testing educational interventions, compare delivery modalities (face-to-face, digital, peer-led) for cost-effectiveness, and validate findings across diverse Saudi regions.

## Conclusion

Disease-specific hypertension knowledge represents a critical, modifiable determinant of self-efficacy among Saudi patients, explaining 25.4% incremental variance beyond demographic and clinical factors. The relationship is significantly moderated by educational attainment (*r* = 0.81 university-educated vs. *r* = 0.59 primary education), supporting the integrated application of Social Cognitive Theory and Health Belief Model in revealing reciprocal relationships among knowledge, environmental contexts, self-efficacy, and health behaviors.

Substantial knowledge deficits (70% unsatisfactory) alongside heterogeneous self-efficacy patterns (35% adequate for exercise vs. 72% for medication adherence) underscore needs for comprehensive, multi-domain educational interventions stratified by literacy level, adapted for age, and sustained throughout the disease course. The robust knowledge-efficacy association, regardless of blood pressure control status, suggests education retains motivational value during difficult treatment phases. As Saudi Arabia confronts high hypertension prevalence with suboptimal control (63% uncontrolled), these insights provide actionable pathways for enhancing self-management through theoretically grounded, empirically informed, contextually tailored patient education strategies.

## Data Availability

The raw data supporting the conclusions of this article will be made available by the authors, without undue reservation.

## References

[1] Mills KT Stefanescu A He J. The global epidemiology of hypertension. Nat Rev Nephrol. (2020) 16:223–37. doi: 10.1038/s41581-019-0244-2 32024986 PMC7998524

[2] Religioni U Barrios-Rodríguez R Requena P Borowska M Ostrowski J. Enhancing therapy adherence: impact on clinical outcomes, healthcare costs, and patient quality of life. Medicina. (2025) 61:153. doi: 10.3390/medicina61010153 39859135 PMC11766829

[3] Nasser SM Shubair MM Fatani F Alhawiti NM Aleissa B Aldubikhi AIS . Prevalence of hypertension and associated factors: a cross-sectional study in Riyadh, Saudi Arabia. BMC Health Serv Res. (2025) 25:351. doi: 10.1186/s12913-025-12481-7 40055707 PMC11887066

[4] Alshammari SA Alshammari AS Alshammari HS Ahamed SS. Overview of hypertension in Saudi Arabia: a systematic review and meta-analysis. Saudi Med J. (2023) 44:951–64. doi: 10.15537/smj.2023.44.10.20230178 37777271 PMC10541986

[5] Dou X Ji M Sun Z Sun H Wang Y Zou H . The impact of psychological factors on hypertension and its psychological intervention in pilot selection candidates. Front Psychol. (2025) 16:1634423. doi: 10.3389/fpsyg.2025.1634423 41280167 PMC12631240

[6] Jareebi MA. The dynamic interplay of lifestyle, dietary factors, and cardiometabolic risk in hypertension: a cross-sectional investigation among Saudi adults. Diagnostics. (2025) 15:2097. doi: 10.3390/diagnostics15162097 40870949 PMC12386032

[7] Burlacu A Kuwabara M Brinza C Kanbay M. Key updates to the 2024 ESC hypertension guidelines and future perspectives. Medicina. (2025) 61:193. doi: 10.3390/medicina61020193 40005310 PMC11857694

[8] Charchar FJ Prestes PR Mills C Ching SM Neupane D Marques FZ . Lifestyle management of hypertension: international society of hypertension position paper endorsed by the world hypertension league and European society of hypertension. J Hypertens. (2024) 42:23–49. 37712135 10.1097/HJH.0000000000003563 PMC10713007

[9] Soliman N Soliman AT. Global strategies for hypertension prevention: evidence from lifestyle and policy interventions (2010–2025). Discov Public Health. (2026) 23:50. doi: 10.1186/s12982-026-01366-7

[10] Allegrante JP Wells MT Peterson JC. Interventions to support behavioral self-management of chronic diseases. Annu Rev Public Health. (2019) 40:127–46. doi: 10.1146/annurev-publhealth-040218-044008 30601717 PMC6684026

[11] Centers for Disease Control and Prevention. Evidence of impact for self-management support and education. Heart Dis Stroke Best Pract Clearingh. (2025). Available online at: https://hdsbpc.cdc.gov/s/article/Evidence-of-Impact-for-Self-Management-Support-and-Education (Accessed November 20, 2025).

[12] Silva JPS Cruz HRA Silva GAG Gualdi LP Lima ÍNDF. Illness perception and self-care in hypertension treatment: a scoping review of current literature. BMC Health Serv Res. (2024) 24:1529. doi: 10.1186/s12913-024-12001-z 39623470 PMC11613738

[13] Bandura A. Health promotion by social cognitive means. Health Educ Behav. (2004) 31:143–64. doi: 10.1177/1090198104263660 15090118

[14] Abboud M Karam S. Hypertension in the Middle East: current state, human factors, and barriers to control. J Hum Hypertens. (2021) 36:428–36. doi: 10.1038/s41371-021-00554-z 34075186

[15] Khormi YH. The health belief model: a framework for understanding health behavior. J Community Public Health Nurs. (2025) 11.

[16] Tan FCJH Oka P Dambha-Miller H Tan NC. The association between self-efficacy and self-care in essential hypertension: a systematic review. BMC Fam Pract. (2021) 22:44. doi: 10.1186/s12875-021-01391-2 33618661 PMC7901221

[17] Han HR Benjasirisan C Metlock FE Tesfai Y Commodore-Mensah Y. Use of the hypertension self-care profile: a scoping review. Int J Environ Res Public Health. (2025) 22:1244. doi: 10.3390/ijerph22081244 40869830 PMC12386562

[18] Al Hazmi AH Alanazi ADM Thirunavukkarasu A Alriwely NS Alrais MMF Alruwaili ABS . Evaluation of hypertension knowledge and its association with medication adherence among hypertensive patients attending primary health centers: a cross-sectional study from eastern Saudi Arabia. Front Public Health. (2025) 12:1378561. doi: 10.3389/fpubh.2024.1378561 39872100 PMC11770004

[19] Salmanpour N Salehi A Nemati S Rahmanian M Zakeri A Drissi HB . The effect of self-care, self-efficacy, and health literacy on health-related quality of life in patients with hypertension: a cross-sectional study. BMC Public Health. (2025) 25:2630. doi: 10.1186/s12889-025-23914-7 40753227 PMC12317574

[20] Huang Y Wang T Wang H Zeng Y Xie L. Health beliefs mediates the association between the number of non-communicable diseases and preventive behaviors in middle-aged and older adults in southern China. Aging Clin Exp Res. (2025) 37:49. doi: 10.1007/s40520-025-02939-3 39994128 PMC11850486

[21] Kam BS Lee SY. Integrating the health belief model into health education programs in a clinical setting. World J Clin Cases. (2024) 12:6660. doi: 10.12998/wjcc.v12.i33.6660 39600476 PMC11514333

[22] Alenazi AM Alqahtani BA. National and regional prevalence rates of hypertension in Saudi Arabia: a descriptive analysis using the national survey data. Front Public Health. (2023) 11:1092905. doi: 10.3389/fpubh.2023.1092905 37081959 PMC10110943

[23] Whelton PK Carey RM Aronow WS Casey DE Collins KJ Dennison Himmelfarb C . 2017 ACC/AHA/AAPA/ABC/ACPM/AGS/APhA/ASH/ASPC/NMA/PCNA guideline for the prevention, detection, evaluation, and management of high blood pressure in adults. J Am Coll Cardiol. (2018) 71:e127–248. doi: 10.1016/j.jacc.2017.11.006 29146535

[24] Callahan CM Unverzagt FW Hui SL Perkins AJ Hendrie HC. Six-item screener to identify cognitive impairment among potential subjects for clinical research. Med Care. (2002) 40:771–81. doi: 10.1097/00005650-200209000-00007 12218768

[25] Faul F Erdfelder E Buchner A Lang AG. Statistical power analyses using G^*^Power 31: tests for correlation and regression analyses. Behav Res Methods. (2009) 41:1149–60. doi: 10.3758/BRM.41.4.1149 19897823

[26] Hair JF Black WC Babin BJ Anderson RE. Multivariate Data Analysis. 8th ed. Mason, OH: Cengage Learning. (2019).

[27] VanVoorhis CRW Morgan BL. Understanding power and rules of thumb for determining sample sizes. Tutor Quant Methods Psychol. (2007) 3:43–50. doi: 10.20982/tqmp.03.2.p043

[28] Asher A Hariharan M. Hypertension knowledge test: development and validation. Int J Indian Psychol. (2017) 5. doi: 10.25215/0501.045

[29] Erkoc SB Isikli B Metintas S Kalyoncu C. Hypertension knowledge-level scale (HK-LS): a study on development, validity and reliability. Int J Environ Res Public Health. (2012) 9:1018–29. doi: 10.3390/ijerph9031018 22690180 PMC3367294

[30] Zhao Y Wang L Li Y Zhang X. Development and validation of the Self-Efficacy for hypertension treatment adherence (SE-HTA) scale. Patient Prefer Adherence. (2021) 15:1231–40.

[31] Polit DF Beck CT. The content validity index: are you sure you know what's being reported? Critique and recommendations. Res Nurs Health. (2006) 29:489–97. doi: 10.1002/nur.20147 16977646

[32] Koo TK Li MY. A guideline of selecting and reporting intraclass correlation coefficients for reliability research. J Chiropr Med. (2016) 15:155–63. doi: 10.1016/j.jcm.2016.02.012 27330520 PMC4913118

[33] Ozoemena EL Iweama CN Agbaje OS Umoke PCI Ene OC Ofili PC . Effects of a health education intervention on hypertension-related knowledge, prevention, and self-care practices in Nigerian retirees: a quasi-experimental study. Arch Public Health. (2019) 77:23. doi: 10.1186/s13690-019-0349-x 31143446 PMC6532220

[34] Hussain MA Mamun AA Reid C Huxley RR. Prevalence, awareness, treatment and control of hypertension in Indonesian adults aged ≥40 years: findings from the Indonesia Family Life Survey (IFLS). PLoS ONE. (2016) 11:e0160922. doi: 10.1371/journal.pone.0160922 27556532 PMC4996427

[35] Liu Y Jiang F Zhang M Niu H Cao J Du S . Health literacy and self-management among middle-aged and young hypertensive patients: a parallel mediation effect of illness perception and self-efficacy. Front Psychol. (2024) 15:1349451. doi: 10.3389/fpsyg.2024.1349451 38765827 PMC11099212

[36] Warner LM Schwarzer R. Self-efficacy and health. In:Liamputtong P, editor *Handbook of Concepts in Health, Health Behavior and Environmental Health*. Singapore: Springer Nature. (2024). doi: 10.1007/978-981-97-0821-5_15-1

[37] Chen TY Kao CW Cheng SM Liu CY. Factors influencing self-care among patients with primary hypertension: path analysis of mediating roles of self-efficacy and depressive symptoms. Eur J Cardiovasc Nurs. (2023) 22:620–7. doi: 10.1093/eurjcn/zvad011 36637099

[38] Kawafha M Alsaqer K Al Maghaireh D Shiyyab H Al Kofahi A Saleh M. The association between health literacy and self-care of hypertension among older adults in five regions in Jordan. Working Older People. (2025) 29:137–45. doi: 10.1108/WWOP-06-2024-0029

[39] González-González E Requena C. Self-care interventions of community-dwelling older adults: a systematic review and meta-analysis. Front Public Health. (2023). doi: 10.3389/fpubh.2023.1254172 37876713 PMC10593480

[40] Li L. Mental health interventions with older adults and the policy implications. Public Policy Aging Rep. (2025) 35:53–6. doi: 10.1093/ppar/praf008

[41] Kühn L Bachert P Hildebrand C Kunkel J Reitermayer J Wäsche H. Health literacy among university students: a systematic review of cross-sectional studies. Front Public Health. (2021) 9:680999. doi: 10.3389/fpubh.2021.680999 35127605 PMC8814326

[42] Kong Y. The role of experiential learning on students' motivation and classroom engagement. Front Psychol. (2021) 12:771272. doi: 10.3389/fpsyg.2021.771272 34744950 PMC8569223

[43] Basileo LD Otto B Lyons M Vannini N Toth MD. The role of self-efficacy, motivation, and perceived support of students' basic psychological needs in academic achievement. Front Educ. (2024) 9:1385442. doi: 10.3389/feduc.2024.1385442

[44] Feng H Luo Z Wu Z Li X. Effectiveness of peer-assisted learning in health professional education: a scoping review of systematic reviews. BMC Med Educ. (2024) 24:1467. doi: 10.1186/s12909-024-06434-7 39695653 PMC11653801

[45] Kilgour AH Rutherford M Higson J Meredith SJ McNiff J Mitchell S . Barriers and motivators to undertaking physical activity in adults over 70—a systematic review of the quantitative literature. Age Ageing. (2024) 53:afae080. doi: 10.1093/ageing/afae080 38651329 PMC11036106

[46] Dwairej L Ahmad M. Hypertension and mobile application for self-care, self-efficacy and related knowledge. Health Educ Res. (2022) 37:199–210. doi: 10.1093/her/cyac012 35582884

